# User testing of a Scottish Intercollegiate Guideline Network public guideline for the parents of children with autism

**DOI:** 10.1186/s12913-021-07384-2

**Published:** 2022-01-15

**Authors:** Naomi Fearns, Laura Walker, Karen Graham, Norman Gibb, Duncan Service

**Affiliations:** Healthcare Improvement Scotland, Gyle Square 1 South Gyle Crescent, Edinburgh, EH12 9EB Scotland UK

**Keywords:** Guidelines, Clinical practice guideline, Patient guidelines, Patient version, Public version, Autism, User testing

## Abstract

**Background:**

The Scottish Intercollegiate Guidelines Network (SIGN) is the leading national clinical guideline producer in Scotland. Improved design and dissemination of guidelines produced for the public can empower people to take an active role in self-management and shared decision-making. The public version of the guideline examined covered getting assessed and diagnosed with autism, and approaches that can help. The aim of this study was to test a public version of a guideline for the parents of children and young people with autism, implement improvements, and identify what works in making it usable and accessible.

**Methods:**

We recruited mothers from across Scotland. User testing involved formal ‘think aloud’ semi-structured interviews that guided users through the booklet. Interviews took place individually and were recorded and transcribed. Key findings were identified and themed using the honeycomb user experience model.

**Results:**

Fourteen user-testing interviews were conducted. Facilitators for usability and desirability of the guideline included the chunking of text, consistent use of colour and boxes to highlight important information. Simple language, written in a tone of partnership, helped to engage mothers. Value arose from the guidelines ability to explain the process of diagnosis and make mothers feel empowered in their relationships with healthcare professionals. There was a lack of consensus on the usefulness of rating the strength of evidence and recommendations.

**Conclusion:**

There was a marked similarity between what was important to the mothers and what has been found to be important to other groups. The involvement of service users and carers in the guidelines development was key to its credibility. One size does not fit all in presenting evidence-based recommendations to the public and it is a challenge to provide sufficient information while avoiding information overload. Recommendations and evidence levels are suitable for use in public versions, but these should be kept as simple as possible.

## Background

Clinical guidelines are tools that provide evidence based recommendations and direct appropriate healthcare [1–3]. Traditionally guidelines are developed to guide clinicians’ decision-making, but there is a growing interest and demand for public versions of clinical guidelines. In the UK, both the Scottish Intercollegiate Guideline Network (SIGN) and the National Institute for Health and Care Excellence (NICE) produce versions of their guidelines for the public. Public versions can help people to participate more actively in their care and engage in shared decision-making [4]. Despite the growing recognition of the value of public versions, an evaluation of guidelines on the resection of hepatocellular carcinoma found that only 2 of 22 guidelines had developed information for the public [5] and a review found just 24% of tools developed to aid the implementation of guidelines were for the public [6].The DECIDE (Developing and Evaluating Communication Strategies to support Informed Decision and practice based on Evidence) project [7] found that awareness of clinical guidelines is low in the general public, and that even when people are aware of them they may not perceive them any more positively than alternative sources of health information [1, 4, 8]. The project concluded that members of the public want actionable information to help them to choose between interventions and that helps them to participate in shared decision-making. “One size does not fit all” in public versions, but it is clear that a guideline must be attractive, easy to use and be available in multiple formats to be accessible and useful to the public [7].

Autism spectrum disorders are a highly prevalent condition. Prevalence in children is around 1% [9, 10]. Swift diagnosis, appropriate interventions, structured support and specialised educational programmes, may help children with autism to fulfil their potential [11–13]. Services across Scotland have adhered to the majority of the recommendations on assessment and diagnosis made in previous SIGN guidelines on autistic spectrum disorders, and it is hoped that this guideline, will continue to have a positive impact on the delivery of services in Scotland [14, 15]

User testing has been used successfully to evaluate and improve systematic reviews, guideline creation tools [16] and dissemination methods with healthcare professionals [17–19] and policy makers [20], as well as the public [8]. User testing identifies fixable flaws in guideline design, which make the guideline inaccessible if they are not corrected [8]. Many questions still remain about how best to format and disseminate guidelines to different groups [16]. Continued user testing of SIGN public versions will help to inform the growing evidence base in this area. The aim of this study was to test and refine a SIGN public version for the parents of children and young people with autism. It also sought to explore how transferable the previous findings of the DECIDE project [1, 4, 8, 21] were to the parents of children with autism.

## Methods

### Participants and setting

Participants were recruited through the SIGN patient network (a ‘virtual’ group of patients, carers, and members of the public), NHS Greater Glasgow and Clyde Child and Adolescent Mental Health Service and from third sector organisations including the National Autistic Society, Autism Network Scotland, and local carers’ centres. Snowball sampling was also used. The SIGN research analyst (LW) contacted organisations, and potential participants were approached by email and telephone. Written information about the project and what participation would involve was provided to all participants prior to the user testing interview. Interviews took place at a location convenient to participants and travel expenses were provided. The names used in quotations are pseudonyms. We aimed to recruit 12 to 15 participants because our previous user testing suggested that this would be sufficient to reach a saturation of views.

Interviews took place in November 2016 in locations across Scotland including Glasgow, Edinburgh, Perth, Dundee and Arbroath.

### User Testing Interviews

User testing was based on a method of data collection and analysis, which was developed by Rosenbaum [17]. This method uses a think-aloud protocol and a semi-structured interview guide, which involved participants being asked to verbalise their thoughts while they read the guideline. This was audio recorded and transcribed. The interview guide was structured around the honeycomb model of user experience, which has six facets: usability, credibility, usefulness/value, desirability, accessibility and findability [22]. Interviews took place individually and face-to-face and lasted approximately one hour.

One interviewer (LW) conducted all the interviews, and a SIGN public partner (a volunteer who works with SIGN to provide a public perspective) acted as an observer and took structured notes. The observer was not available for the final three interviews. A training session was held with the observer prior to the start of data collection. A pilot interview was held to examine the feasibility of the interview guide, and resulted in minor amendments, including the order of the questions, and the print quality of materials.

### The guideline

There are three public versions of SIGN guideline 145: Assessment, diagnosis and interventions for autism spectrum disorders [23], a booklet for young people with autism; one for adults with autism; and one for parents, carers and families. The user testing examined a draft of the public version for parents, carers and families. The draft was designed on the principles identified in our previous publications [4, 8]. Following the user testing, improvements were made to the guideline [24]

SIGN’s public versions used three pre-defined levels of evidence, to explain the evidence quality underlying our advice to the public. We tested the use of an additional level of evidence, for a strong recommendation based on good quality evidence (see Fig. 3). The new format matches more closely with the evidence levels used in SIGN’s clinical guidelines [25]. SIGN’s levels of evidence are based on GRADE recommendations [26, 27]

SIGN public versions aim to be an accurate translation of the clinical guideline and only those interventions recommended in the guideline are included. However, further information is included to help the public to understand the recommendations.

### Analysis

The method of analysis is described in detail in our previous paper [8]. It incorporates two phases:

1. phase one identified barriers to the use of the public version and necessary amendments to the guideline and was published on SIGNs website [24],
2. phase two identified issues that may be generalisable beyond this specific guideline, and is reported on here.

In phase two, the findings were refined and analysed thematically [28] using the honeycomb user experience model [22] as a framework. Two researchers (LW and NF) carried out the thematic analysis for phase two, using NVivo® 10. The honeycomb model of user experience provided a theory to inform the analysis and an overarching thematic framework. The researchers familiarised themselves with all the transcripts. All transcripts were then coded in their entirety into meaningful chunks of text by one researcher (LW). Themes were then derived from these codes. At this point, a second researcher (NF) independently coded a purposive sample of three transcripts from different regions of Scotland.

The researchers met to discuss the results, their discussions were focused on identifying areas of agreement and disagreement and on reaching consensus on their interpretation of participants’ interview transcripts. Agreement was reached on key themes and findings, no statistical form of inter-rater reliability was used [29].

The lower order themes were then grouped under the overarching themes from the honeycomb model. An iterative process of writing up the findings and refining the themes was then carried out, which involved both researchers [28, 30]

The researchers recognise the need for substantial analytical and interpretative work on the part of qualitative researchers and acknowledge that coding is a subjective process. However, the research values, experience, skills and training of qualitative researchers and the application of rigorous research and analytical methods should ensure that the themes and findings generated are authentic and trustworthy [30]

### Ethics

This project is classified as a NHS service evaluation. Information on the planned data collection was submitted to the East of Scotland Research Ethics Committee, who confirmed that full ethical approval was not required. The project was carried out in accordance with the principles laid out in the declaration of Helsinki. Participants were sent an information sheet in advance, and gave informed written consent before their interview began. Participants were informed of their right to withdraw at any point of the study, all data was held securely and no personally identifiable information or quotes have been published.

## Results

### Participants

Fourteen mothers of people with autism took part in the study. Sixteen people agreed to take part but two people were unable to make the available dates. All of the participants were female, with a mean age of 42 and a range of 10 years, they were largely well educated, and involved in a range of activities (such as volunteering) in relation to autism, see Table 1

**Table 1:** participant information

Participant pseudonym	Gender	Level of education	Source recruited from
Jennifer	Female	In college level further education	SIGN patient network
Ruth	Female	Secondary school qualifications	Snowballing
Susan	Female	Secondary school qualifications	Carers Centre
Lisa	Female	Undergraduate degree	SIGN patient network
Angela	Female	Postgraduate degree	CAMHS
Diane	Female	Higher National Diploma (HND)	Carers Centre
Carol	Female	Postgraduate degree	Snowballing
Karen	Female	Undergraduate degree	SIGN Facebook
Michelle	Female	Secondary school qualifications	Snowballing
Patricia	Female	In college level further education	SIGN patient network
Jill	Female	Undergraduate degree	SIGN Facebook
Rachel	Female	Undergraduate degree	Carers Centre
Mary	Female	Higher degree	SIGN Facebook
Heather	Female	Postgraduate degree	SIGN patient network

### Findings

### Usefulness and value

There was agreement from most mothers that the guideline would be a valuable resource for families of children with autism, particularly at the time of assessment and diagnosis. The parts of the guideline that mothers found most useful included, information on their child’s health condition, tools and tests used for assessment, information on interventions, what to expect from healthcare professionals and services at each stage of diagnosis, and signposting to sources of information and support.

**Fig. 1:**
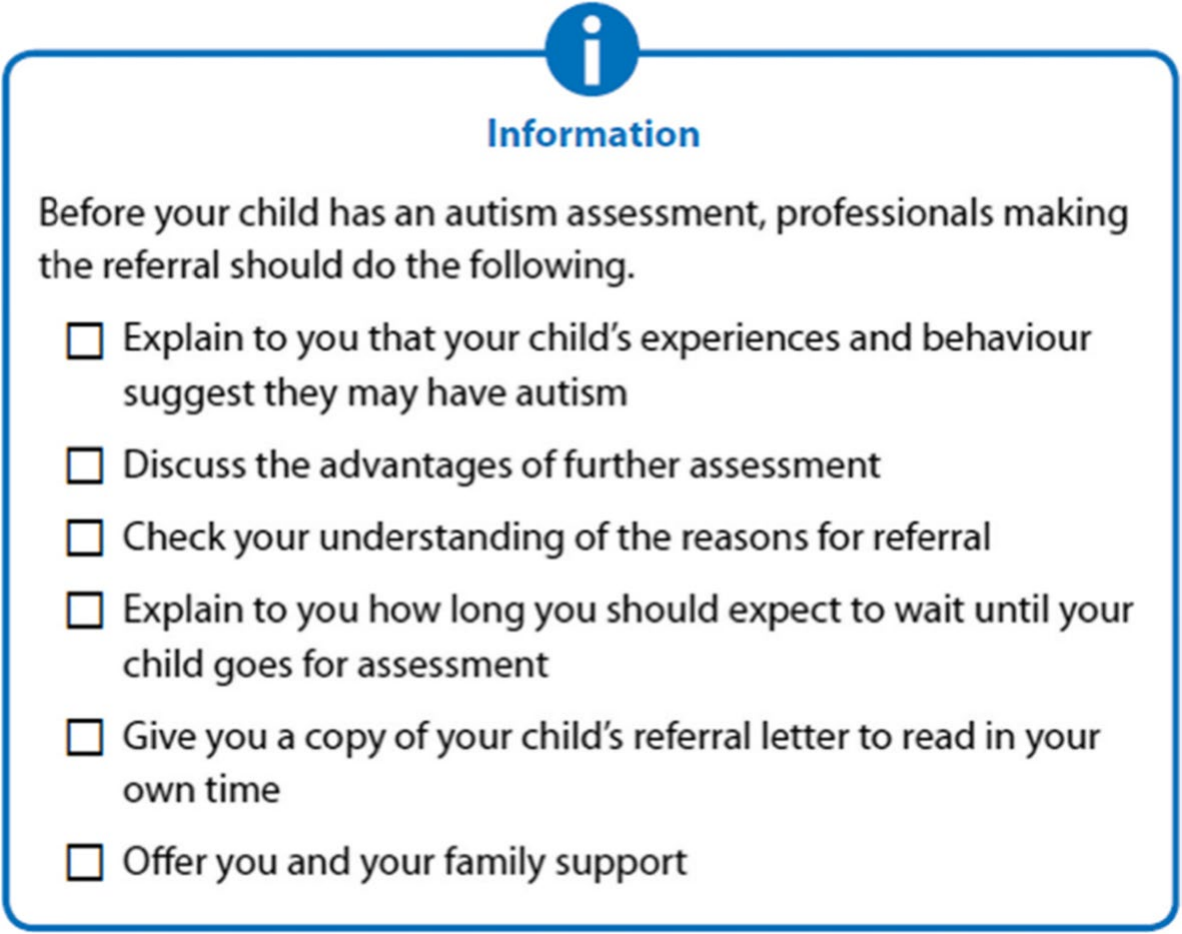
information box

Mothers suggested that the public version empowered them to ask informed question of healthcare professionals and to participate in shared decision-making. Information was valued, even if it was not linked to a recommendation, because it could be used in discussions with healthcare professionals and to highlight specific services or interventions (see Fig. 1). This type of information was most useful when it contained ‘tick boxes’, or other interactive tools.

“… it [Fig. 1] tells you what the professional should be doing…I would probably refer back and say “well, I don’t think we’ve discussed X, Y or Z, so can you give me some more information about that”…[it] is a daunting process, for anyone, so the more information you can have the better.” Rachel

The ‘Where can you find out more?’ section provided a list of trusted sources of help and information. Mothers wanted local support information, but this could not be provided in a national guideline. Signposting to national and third sector organisations may be the best way that a national body can meet this need.

### Usability

**Fig. 2:**
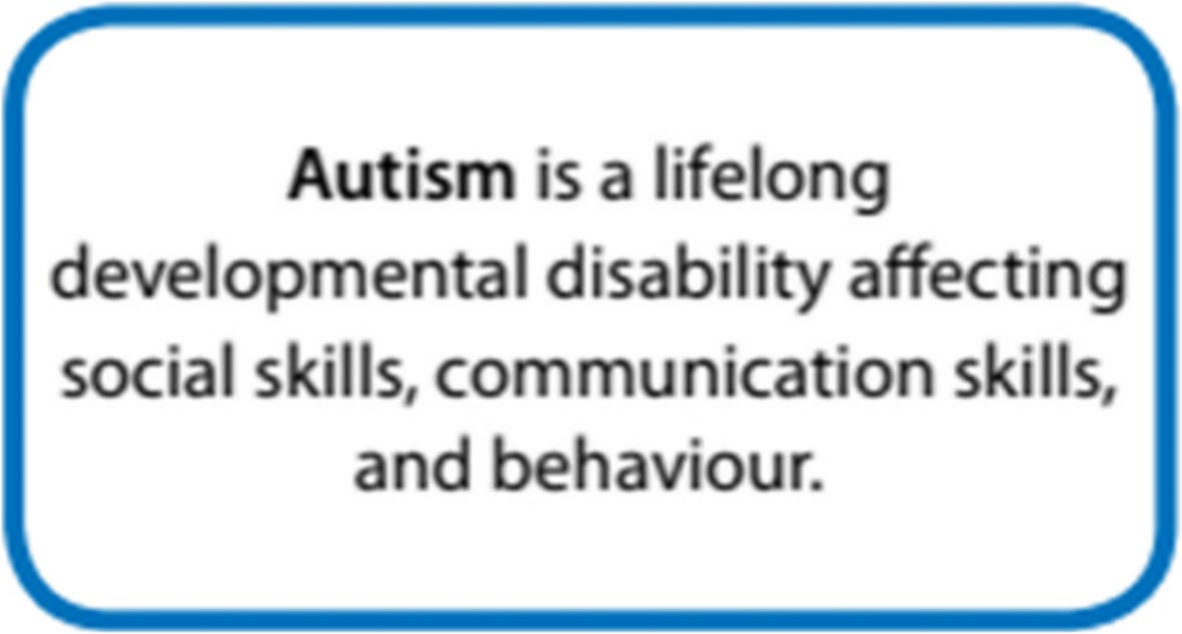
text box

The number of text boxes (see Fig. 2) used in this version, sometimes three or more on a single page was confusing for mothers. Chunking [31] via a mixture of simple text, bullet points and boxes or icons on each page breaks up the text in a more usable way.

Mothers highlighted the importance of the consistent and meaningful use of colour. In SIGN public versions, recommendations are green, which is associated with safety or ‘go’. Information boxes are highlighted in blue (see Fig. 1), which mothers linked with information road signs. Inconsistencies in the use of colour, such as too many coloured boxes on one page, or text that seemed unrelated appearing in the same colour boxes, was overwhelming for some mothers.

**Fig. 3:**
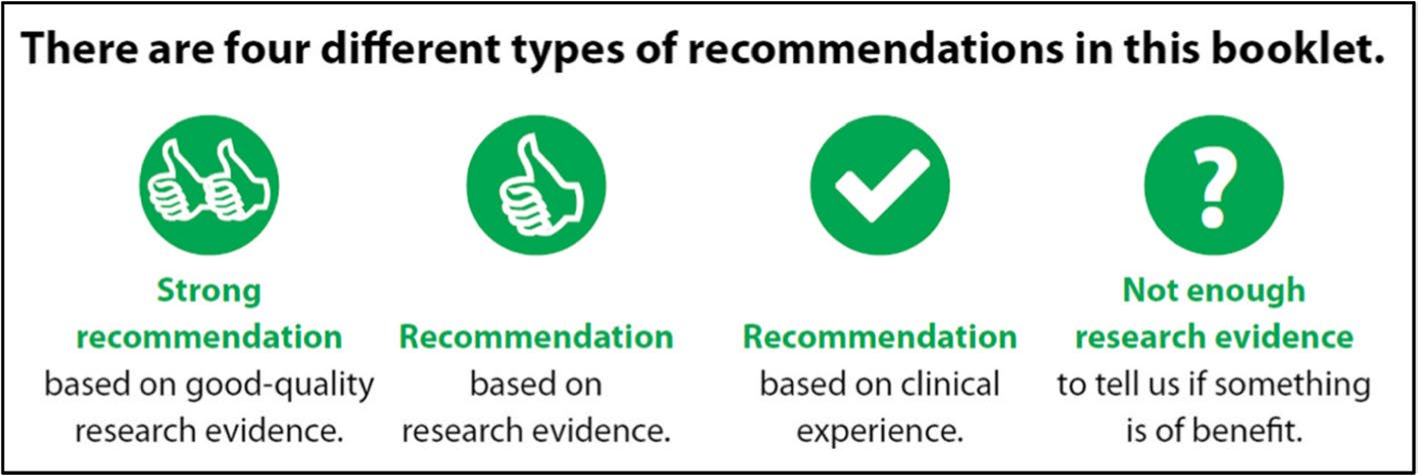
recommendation levels

SIGN uses a system of icons with text to flag recommendations and their evidence level (see Fig. 3). Mothers found the use of thumbs up, tick and question mark symbols clear and easy to understand. However, the response to the underlying four levels of evidence was mixed. Some mothers appreciated the level of detail offered by the grades, and others thought it would be sufficient simply to know that SIGN recommended an intervention.

“I think, I suppose it’s saying ‘its ok’, - based on research, based on clinical. I would say probably your Joe Public will just think it’s one in the same thing, they’re not going to be differentiating it at all.” Diane

While the mothers understood the essential message of the evidence levels, that one intervention is strongly recommended and another one less strongly recommended, most did not understand *why* it is necessary to have these different levels of recommendation. Similarly, mothers found the use of the not enough evidence icon (see Fig. 3) disconcerting. While they understood that the question mark and text was meant to convey uncertainty, they did not like this message, or understand why guideline producers would need to use it.

“…to the lay person….what makes one good quality you know if it’s basing it on evidence surely it should be of a certain quality otherwise why would you use that recommendation…surely all research should be good quality and if not why not?” – Michelle.

## Credibility

**Fig. 4:**
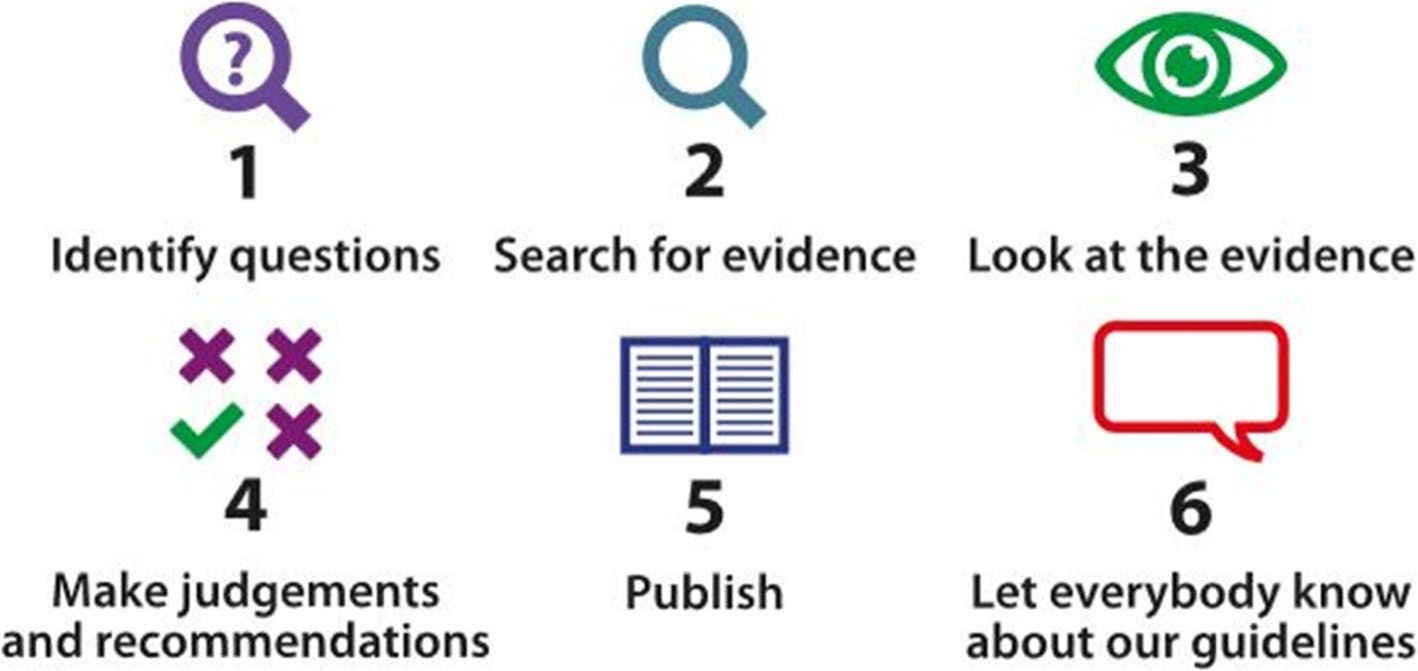
how SIGN develop their guidelines

Mothers valued the public version because they saw it as a trusted and credible source of information. Its credibility arose from the simple and clear description of the process used in the development of the guideline (See Fig. 4) and the evidence-based nature of the recommendations.

“… read it [Fig. 4] and know that that’s how the process has been gone through and the information that is there has been, like accredited, if you like and you’d feel confident that it had gone through all the relevant stages to know that that’s the most up-to-date information to use.”—Rachel.

The simple information provided on the process of guideline development reassured the mothers that the public version was based on up-to-date evidence. While some people would have preferred more detailed information, this was sufficient for the majority of mothers, and considered too much information by some.

Service user and carer input in the guideline development process was key to the relevance and credibility of the recommendations. Service users and carers are involved in SIGN’s guideline development process, including representation on the guideline development group, and this is mentioned in the information provided in the public version. However, this information was not salient enough for all the mothers and needed to be made more detailed and prominent.

Jill describes below, how it would have been reassuring if a larger group of carers and people with autism had been even more actively involved in the guideline development process to ensure that the included information was not too medical in orientation and was meaningful and actionable for carers. 

"I kind of glossed over that [key to levels of recommendation/evidence] to be honest…Ok so there are four different types of recommendation, and it’s SIGNs recommendations. Yeah, it’s clear, but I don’t really know who SIGN is so I don’t know if I trust their opinion. Whereas if you had a body of parents and carers…who have given their thumbs up, double thumbs up, question mark or ‘dubious about that, cause that’s what the doctors say but it’s nonsense in real life’ that would probably hold more weight for me personally…"

– Jill.

Concerns regarding the status or enforceability of the guideline were evident. Mothers wanted to be able to use it to discuss their options with healthcare professionals and to make a case for receiving the recommended interventions. Therefore they wanted to know if the guidelines were enforceable and what they could do if their children’s’ care was not in line with the recommendations.

"…I see all the recommendations, and realise that actually, it doesn’t happen… if you read this, and this isn’t happening, what can you do?…because [it is] all very well having the guidelines, but if they’re not being adhered to where does that leave you as a parent, can you just wander down and go ‘here, I’ve got these guidelines’ and they can say well actually I don’t have to do that…."

Michelle

## Desirability

For the mothers of children with autism, the use of inclusive and sensitive language and terminology was especially important. The tone should be collaborative, positive and reassuring; however, this must be balanced by the need to convey information clearly, some of which may be perceived as negative or ‘scary’.

…a lot of the language I like, because in many things they talk very much about autism as ‘they’ as if they’re some sort of strange distant individual. It seems much more collaborative…especially the bit where it talks about occupational therapist that they’re people who can ‘help’ I think that’s very good language, because sometimes it’s the word “treat”…

Karen

Terms used to describe people with autism, provoked a strong emotional response. Mothers preferred terms that that they recognised, from their child’s diagnosis, from service user information sites or social media pages for carers. They had different preferences such as ‘autistic person/child’, ‘with autism’ or ‘having autism’. Another example of emotive language was the term 'anti-psychotic', used in a recommendation about medication. Additional contextual information, which acknowledges some of these issues, might help to make this recommendation less off-putting.

"…a lot of people see the word anti-psychotic and go ‘why are you giving my child antipsychotics, they’re not psychotic, what’s going on’…a little bit more background as to it’s a particular kind of medicine that is used for certain behaviours and don’t let the word scare you… you know so that’s a bit vague…"

- Mary.

The inclusion of quotes from parents helped to personalise the material and make the information less medical in tone. Mothers felt they could relate to the quotes, and that they helped to normalise their fears around diagnosis. Quotes also posed a potential risk to the desirability and accessibility of the booklet because what may personalise the material for one person may feel alienating to another. Mothers of girls may feel excluded by the majority of quotes referring to boys names. Some mothers liked the supportive or emotional tone of the quotes while others would have appreciated a more practical orientation to them.

"….I think the ones on page 5, as I said, about gender when you see two quotes, both of boys again you think “oh here we go”…maybe just a few quotes about how you would get through the medical process or “yes the waiting times are long, but …” that sort of thing. I think a lot of the quotes are sort of emotional quotes as opposed to maybe practical quotes."– Karen.

### Accessibility and findability

Mothers highlighted the need to have the public versions available in multiple formats to maximise accessibility and findability. This included availability online and as hard copies. Availability online helps people to find the guideline and makes sharing the booklet with peers easier. Making hard copies of it available makes it accessible to people who do not routinely have access to the internet, and allows distribution directly by healthcare professionals, which was the strongest preference for distribution expressed by the mothers.

"…it would be even more appealing if the practitioners…introduced it to the family…it could be available in for example things like that first developmental assessment…because you do go away with nothing generally. And then the risk is a lot of people resort to doing Google searches and getting all sorts of stuff." – Angela.

A potential barrier to mothers finding and using this public version was confusion about its exact purpose. The SIGN logo and accompanying text saying ‘Scottish guidelines’ was printed on the front cover and more information on who the booklet is designed for was given on page 2. Despite this, it was not clear to all of the mothers that this was a version of a clinical guideline that had been designed specifically for parents and carers of children with autism. This can lead to confusion about why this booklet would be helpful for them and how and when they would be able to access it.

## Discussion

The points of key importance about the credibility, usability, findability and desirability of this public versions of a clinical guideline for parents and carers of children and young people with autism, were similar to those identified by the DECIDE project in other groups [4, 8]. These included the chunking of text [31], consistent use of colour, the use of boxes to highlight important information and simple language using a collaborative tone. Some issues and preferences about the design of the public version were more prominent in the mothers of children with autism, including the involvement of carers and people with autism in SIGNs processes.

Many of the suggestions made by mothers led to changes to the final version of the booklet [24]. For example, the font size was increased on the banner on the front page; the title was amended to include the word ‘families’; and a sentence thanking parents and carers that contributed to the consultation of the booklet was moved to the inside cover to make their involvement more salient. A section on the range of terms for autism and how SIGN chose their terminology was added, it noted that SIGN use the term autism but are aware that parents have different preferences. The final version of this guideline, which incorporated the results of this user testing is available on SIGN’s website [32]

Consistent with previous research [4, 8, 33] the usefulness of the public version was dependant on providing credible, evidence-based information, that mothers felt empowered them to navigate the healthcare system and get involved in shared decision-making. A substantial amount of contextual information, not directly linked to the recommendations, may be required to facilitate this, and guideline makers must strike a balance between including all potentially useful information and brevity. At 58 pages the length of the booklet [32] may be off-putting for some members of the public [4, 8, 34]

There is a lack of public access to clearly trustworthy information about the effects of health interventions online [35]. Public versions of clinical guidelines might be well placed to fill this gap in the provision of evidence based accessible healthcare information. Unfortunately, awareness of clinical guidelines and the availability of public versions of clinical guidelines is low in the general public, and evidence producers websites may not be easily found or user friendly [1, 4, 17]. Simple publication on guideline makers’ websites is unlikely to be sufficient to disseminate information to the public [33]. The general preference of the public seems to be for distribution directly by healthcare professionals, however it is also important that public versions are accessible online [4, 8]. Guideline makers should consider options, such as forming partnerships with third sector organisations, who may aid dissemination by linking to public versions of relevant guidelines, or making use of other relevant portals [33]. 

A lack of awareness about the existence of lower quality research, and why a guideline producer would need to use it, leads to some members of the public not valuing (or understanding) the need for different levels of recommendation. When involving the public in guideline development, training can be offered about evidence quality [36] to explain their use, however, this presents a much more challenging problem for writing  public versions of guidelines. Much material created for the public based on clinical guidelines sidesteps this problem by not referring directly to levels of evidence or recommendations [36]. Variation in the public’s appetite for detail about the evidence that underlies recommendations is a difficult challenge to overcome in printed materials, although layering of information in electronic resources is helpful [37]

Work in the field is ongoing on developing quality criteria for public versions of clinical guidelines and other decision aids [38–40], and this research makes clear the importance of ensuring that public versions reflect the relevant recommendations and that recommendations are clearly flagged and not lost within an excess of other information. The GIN public toolkit [36] supports the inclusion of recommendation and evidence grades within public versions, provided that they are used alongside intuitively understandable icons (like the SIGN ticks and thumbs up symbols).

On balance, our user testing supported the use of multiple levels of recommendation. This group understood the meaning of the hierarchy of strength, and recent research supports this in other groups [34]. However, recommendation levels must be kept as simple as possible and it should be clear how people can apply the recommendation to their healthcare context. It may also be helpful to be flexible in the application of graded recommendations and evidence levels and use a simpler approach, when making public versions for people that may have lower reading ages, lower levels of health literacy [41, 42], or shorter attention spans. Where resources allow, multiple formats of public versions should be made available [4, 36]. Public versions of SIGN guidelines are made available in multiple languages and SIGN review individual requests for additional translations upon request; a video of the autism public version in British sign language is also available.

Interestingly the mothers of children with autism placed a greater emphasis on the active inclusion of the public in developing the clinical guideline and on the inclusion of up-to-date evidence than people with glaucoma [8]. Mothers wanted to know that the recommendations would be considered practical and useful by carers, whereas the people with glaucoma placed a stronger emphasis on the knowledge of the healthcare professionals involved in the guideline development. This may be related to the age of the two groups, the glaucoma group consisted largely of older adults who tend to prefer a more directive approach to shared decision-making [43]. Stigma associated with the diagnosis of autism [44], and the role of the mothers in caring and advocating for their children may also have contributed to this preference. Many of the participants were active in the autistic community and used social media sites and other methods to connect with other parents and strongly valued the views and resources shared by this community. Community engagement strategies are one methods of making guidelines more person centred and may be particularly useful for groups such as the parents of children with autism [45]

Unsurprisingly the use of a tone of partnership and collaboration in the public version was important for these mothers. Guideline developers should tailor the language used in public versions to the specified user group as far as possible, and user testing or some other form of consultation is certainly helpful. Acknowledging any challenges with terminology and describing how chosen terms have been selected in the public version, may also reassure the user group that the guideline maker is aware of the sensitivity and controversy that may be associated with them.

SIGN uses consultation with service users and the public to select their recommendations for inclusion in their public versions; however, the public may still struggle to understand how to make practical use of all recommendations included. For example, recommendations that use the question mark symbol to reflect uncertainty in the evidence base. The inclusion of qualitative evidence examining public perspectives in the guideline development process may help to make recommendations more feasible, acceptable and actionable. [46, 47] SIGN have begun to explore how they can incorporate qualitative research more actively into their guidelines, in SIGN 159: Epilepsy in children and young people – diagnosis and management (still in development) [48] and with the recent publication of their first rapid qualitative synthesis, carried out to inform a guideline on foetal alcohol syndrome [49]

## Strengths and limitations

SIGN’s public versions of clinical guidelines were  redesigned based on the work of the DECIDE project between 2011 and 2015 [7]. This study built on our previous user testing by using a guideline that incorporated recommendations about interventions and was targeted at a different user group to those previously studied. This allowed us to explore how transferable our previous findings were to the mothers of children and young people with autism.

The sample was limited to women, all of whom were mothers of a child with autism. The sample consisted mainly of highly educated women, many of whom were active in the autistic community and half of the sample was recruited from SIGNs patient network or via SIGNs Facebook page. This group was more aware of clinical guidelines than the general public and may have held stronger views on aspects of the management of autism than a more diverse sample would. Recruiting from other sources, such as online communities independent from SIGN, or diverse third sector organisation might help to broaden SIGNS samples in future research.

This guideline did not contain numerical information on the risks and benefits associated with intervention options [50]. Future work could explore the publics’ needs concerning this type of information by carrying out user testing with an appropriate SIGN guideline.

## Conclusion

Many aspects of usability, usefulness, desirability and credibility were the same for the mothers of children with autism as for other public groups [8, 4]. The involvement of service users and carers in the development of the guideline and a tone of partnership in the text was particularly important to credibility for the mothers. One size does not fit all in presenting evidence-based recommendations to the public and it is a challenge to provide sufficient information to make people feel empowered while avoiding information overload. Recommendation and evidence levels should be included in most public versions, but kept as simple as possible. Guideline makers should explore multiple methods of including public perspectives in their recommendations to make them more credible to the public and feasible to implement. This may include the use of user-testing, incorporating qualitative research in the guideline development process, and community engagement strategies; as well as more common methods like public consultation and public representatives on guideline committees.

## Data Availability

The datasets generated and analysed during the current study are not publicly available due to the authors **not** having obtained consent from the participants to share their interview transcripts, but are available from the corresponding author on reasonable request and with permission of the interviewees. DECIDE (Developing and Evaluating Communication strategies to support Informed Decision and practice based on Evidence) was funded by the European Union Seventh Framework Programme (Fp7/2007–2013) under grant agreement number 258583.

## Abbreviations

DECIDE: Developing and Evaluating Communication strategies to support Informed Decision and practice based on Evidence
GIN: Guidelines International Network
NICE: National Institute of Clinical Excellence
SIGN: Scottish Intercollegiate Guidelines Network
